# Human Activity Recognition in AAL Environments Using Random Projections

**DOI:** 10.1155/2016/4073584

**Published:** 2016-06-20

**Authors:** Robertas Damaševičius, Mindaugas Vasiljevas, Justas Šalkevičius, Marcin Woźniak

**Affiliations:** ^1^Department of Software Engineering, Kaunas University of Technology, LT-51368 Kaunas, Lithuania; ^2^Institute of Mathematics, Faculty of Applied Mathematics, Silesian University of Technology, 44-100 Gliwice, Poland

## Abstract

Automatic human activity recognition systems aim to capture the state of the user and its environment by exploiting heterogeneous sensors attached to the subject's body and permit continuous monitoring of numerous physiological signals reflecting the state of human actions. Successful identification of human activities can be immensely useful in healthcare applications for Ambient Assisted Living (AAL), for automatic and intelligent activity monitoring systems developed for elderly and disabled people. In this paper, we propose the method for activity recognition and subject identification based on random projections from high-dimensional feature space to low-dimensional projection space, where the classes are separated using the Jaccard distance between probability density functions of projected data. Two HAR domain tasks are considered: activity identification and subject identification. The experimental results using the proposed method with Human Activity Dataset (HAD) data are presented.

## 1. Introduction

The societies in the developed countries are rapidly aging. In 2006, almost 500 million people worldwide were 65 years of age or older. By 2030, that total number of aged people is projected to increase to 1 billion. The most rapid increase of aging population occurs in the developing countries, which will see a jump of 140% by 2030 [1]. Moreover, the world's population is expected to reach 9.3 billion by 2050 [2], and people who are above 60 years old will make up 28% of the population. Dealing with this situation will require huge financial resources to support the ever-increasing living cost, where human life expectancy is expected to reach 81 years by 2100.

As older people may have disorders of body functions or suffer from age-related diseases, the need for smart health assistance systems increases each year. A common method of monitoring geriatric patients is a physical observation, which is costly, requires a lot of human staff, and is increasingly infeasible in view of massive population aging in the following years. Many Ambient Assisted Living (AAL) applications such as care-providing robots, video surveillance systems, and assistive human-computer interaction technologies require human activity recognition. While the primary users of the AAL systems are of course the senior (elderly) people, the concept also applies to mentally and physically impaired people as well as people suffering from diabetes and obesity, who may need assistance at home, and people of any age interested in personal fitness monitoring. As a result, the sensor-based real-time monitoring system to support independent living at home has been a subject of many recent research studies in human activity recognition (HAR) domain [3–10].

Activity recognition can be defined as the process of how to interpret sensor data to classify a set of human activities [11]. HAR is a rapidly growing area of research that can provide valuable information on health, wellbeing, and fitness of monitored persons outside a hospital setting. Daily activity recognition using wearable technology plays a central role in the field of pervasive healthcare [12]. HAR has gained increased attention in the last decade due to the arrival of affordable and minimally invasive mobile sensing platforms such as smartphones. Smartphones are innovative platforms for HAR because of the availability of different wireless interfaces, unobtrusiveness, ease of use, high computing power and storage, and the availability of sensors, such as accelerometer, compass, and gyroscope, which meet the technical and practical hardware requirements for HAR tasks [13–15]. Moreover, technological development possibilities of other applications are still arising, including virtual reality systems. Therefore, these machines present a great possibility for the development of innovative technology dedicated for the AAL systems.

One of the key motivating factors for using mobile phone-based human activity recognition in the AAL systems is the relationship and correlation between the level of physical activity and the level of wellbeing of a person. Recording and analysing precise information on the person's activities are beneficial to keeping the progress and status of the disease (or mental condition) and can potentially improve the treatment of person's conditions and diseases, as well as decreasing the cost of care. Recognizing indoor and outdoor activities such as walking, running, or cycling can be useful to provide feedback to the caregiver about the patient's behaviour. When following the daily habits and routines of users, one can easily identify deviations from routines, which can assist the doctors in diagnosing conditions that would not be observed during routine medical examination. Another key enabler of the HAR technology is the possibility of providing independent living for the elderly as well as for patients with dementia and other mental pathologies, which could be monitored to prevent undesirable consequences of abnormal activities. Furthermore, by using persuasive techniques and gamification, HAR systems can be designed to interact with users to change their behaviour and lifestyles towards more active and healthier ones [16].

Recently, various intelligent systems based on mobile technologies have been constructed. HAR using smartphones or other types of portable or wearable sensor platforms has been used for assessing movement quality after stroke [17], such as upper extremity motion [18], for assessing gait characteristics of human locomotion for rehabilitation and diagnosis of medical conditions [19], for postoperative mobilization [20], for detecting Parkinson's disease, back pain, and hemiparesis [21], for cardiac rehabilitation [22], for physical therapy, for example, if a user is correctly doing the exercises recommended by a physician [23, 24], for detecting abnormal activities arising due to memory loss for dementia care [25, 26], for dealing with Alzheimer's [27] and neurodegenerative diseases such as epilepsy [28], for assessment of physical activity for children and adolescents suffering from hyperlipidaemia, hypertension, cardiovascular disease, and type 2 diabetes [29], for detecting falls [30, 31], for addressing physical inactivity when dealing with obesity [32], for analysing sleeping patterns [33], for estimating energy expenditures of a person to assess his/her healthy daily lifestyle [34], and for recognizing the user's intent in the domain of rehabilitation engineering such as smart walking support systems to assist motor-impaired persons and the elderly [35].

In this paper, we propose a new method for offline recognition of daily human activities based on feature dimensionality reduction using random projections [36] to low dimensionality feature space and using the Jaccard distance between kernel density probabilities as a decision function for classification of human activities.

The structure of the remaining parts of the paper is as follows.  Section 2 presents the overview of related work in the smartphone-based HAR domain with a particular emphasis on the features extracted from the sensor data.  Section 3 describes the proposed method.  Section 4 evaluates and discusses the results. Finally, Section 5 presents the conclusions and discusses future work.

## 2. Overview of HAR Features and Related Work

All tasks of the HAR domain require correct identification of human activities from sensor data, which, in turn, requires that features derived from sensor data must be properly categorized and described. Next, we present an overview of features used in the HAR domain.

### 2.1. Features

While numerous features can be extracted from physical activity signals, increasing the number of features does not necessarily increase classification accuracy since the features may be redundant or may not be class-specific:

- Time domain features (such as mean, median, variance, standard deviation, minimum, maximum, and root mean square, applied to the amplitude and time dimensions of a signal) are typically used in many practical HAR systems because of being less computationally intensive; thus, they can be easily extracted in real time.
- Frequency-domain features require higher computational cost to distinguish between different human activities. Thus, they may not be suitable for real-time AAL applications.
- Physical features are derived from a fundamental understanding of how a certain human movement would produce a specific sensor signal. Physical features are usually extracted from multiple sensor axes, based on the physical parameters of human movements.

Based on the extensive analysis of the literature and features used by other authors (esp. by Capela et al. [17], Mathie et al. [37], and Zhang and Sawchuk [38]), we have extracted 99 features of data, which are detailed in Table 1.

### 2.2. Feature Selection

Feature selection is the process of selecting a subset of relevant features for use in construction of the classification model. Successful selection of features allows for simplification of models to make them easier to interpret, to decrease model training times, and to better understand difference between classes. Using feature selection allows removing redundant or irrelevant features without having an adverse effect on the classification accuracy. There are four basic steps in a typical feature selection method [58]: generation of candidate feature subset, an evaluation function for feature candidate subset, a generation stopping criterion, and a validation procedure.

Further, we analyse several feature selection methods used in the HAR domain.

ReliefF [59] is a commonly used filter method that ranks features by weighting them based on their relevance. Feature relevance is based on how well data instances are separated. For each data instance, the algorithm finds the nearest data point from the same class (hit) and nearest data points from different classes (misses).

Matlab's Rankfeatures ranks features by a given class separability criterion. Class separability measures include the absolute value of a statistic of a two-sample *t*-test, Kullback-Leibler distance, minimum attainable classification error, area between the empirical Receiver Operating Characteristic (ROC) curve and the random classifier slope, and the absolute value of the statistic of a two-sample unpaired Wilcoxon test. Measures are based on distributional characteristics of classes (e.g., mean, variance) for a feature.

Principal component analysis (PCA) is the simplest method to reduce data dimensionality. This reduced dimensional data can be used directly as features for classification. Given a set of *N* features, a PCA analysis will produce new data variables (PCA components) as linear combinations of the features with the highest variance in the subspace orthogonal to the preceding PCA component. As variability of the data can be captured by a relatively small number of PCs, PCA can achieve high level of dimensionality reduction. Several extensions of the PCA method are known such as kernel PCA, sparse PCA, and multilinear PCA.

Correlation-based Feature Selection (CFS) [60] is a filter algorithm that ranks subsets of features by a correlation-based heuristic evaluation function. A feature is considered to be a good one if it is relevant to the target concept but is not redundant to any of the other relevant features. Goodness of measure is expressed by a correlation between features, and CFS chooses the subset of features which has the highest measure. The chosen subset holds the property that features inside this subset have high correlation with the class and are unrelated to each other.

Table 2 summarizes the feature selection/dimensionality reduction methods in HAR.

A comprehensive review of feature selection algorithms in general as well as in the HAR domain can be found in [58, 61–63].

### 2.3. Summary

Related work in the HAR domain is summarized in Table 3. For each paper, the activities analysed, types of sensor data used, features extracted, classification method applied, and accuracy achieved (as given by the referenced papers) are given.

## 3. Method

### 3.1. General Scheme

The typical steps for activity recognition are preprocessing, segmentation, feature extraction, dimensionality reduction (feature selection), and classification [24]. The main steps of activity recognition include (a) preprocessing of sensor data (e.g., denoising), (b) feature extraction, (c) dimension reduction, and (d) classification. The preprocessing step includes noise removal and representation of raw data. The feature extraction step is used to reduce large input sensor data to a smaller set of features (feature vector), which preserves information contained in the original data. The dimensionality reduction step can be applied to remove the irrelevant (or less relevant) features and reduce the computational complexity and increase the performance of the activity recognition process. The classification step is used to map the feature set to a set of activities.

**Figure 1:**
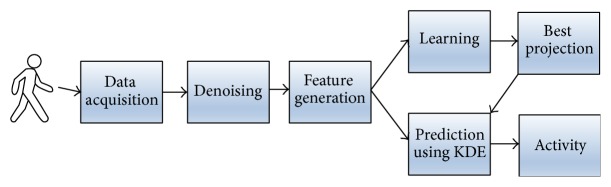
General scheme of the proposed method.

In this paper, we do not focus on data preprocessing and feature extraction but rather on dimensionality reduction and classification steps, since these two are crucial for further efficiency of AAL systems. The proposed method for human activity recognition is based on feature dimensionality reduction using random projections [36] and classification using kernel density function estimate as a decision function (Figure 1).

### 3.2. Description of the Method

During random projection, the original *d*-dimensional data is projected to a *k*-dimensional (*k* ≪ *d*) subspace using a random *k* × *d* matrix *R*. The projection of the data onto a lower *k*-dimensional subspace is *X*_*k*×*N*_^*RP*^ = *R*_*k*×*d*_ *X*_*d*×*N*_, where *X*_*d*×*N*_ is the original set of *N* *d*-dimensional observations. In the derived projection, the distances between the points are approximately preserved, if points in a vector space are projected onto a randomly selected subspace of suitably high dimension (see the Johnson-Lindenstrauss lemma [64]). The random matrix *R* is selected as proposed by Achlioptas [36] as follows:

(1) $$\begin{array}{c} r_{ij}=\left\{\begin{array}{cc} +1, & \text{probability }\frac{1}{6}\\ 0, & \text{probability }\frac{2}{3}\\ -1, & \text{probability }\frac{1}{6}. \end{array}\right. \end{array}$$

Given the low dimensionality of the target space, we can treat the projection of low-dimensional observations onto each dimension as a set of random variables for which the probability density function (PDF) can be estimated using kernel density estimation (KDE) (or Parzen window) method [65].

If *x*_1_, *x*_2_,…, *x*_*N*_ is a sample of a random variable, then the kernel density approximation of its probability density function is

(2) $$\begin{array}{c} {\hat{f}}_{h}\left(\;x\;\right)=\frac{1}{Nh}K\left(\;\frac{x-x_{i}}{h}\;\right), \end{array}$$

where *K* is some kernel and *h* is the bandwidth (smoothing parameter). *K* is taken to be a standard Gaussian function with mean zero and variance 1 of the examined data features:

(3) $$\begin{array}{c} K\left(\;x\;\right)=\frac{1}{\sqrt{2\pi }}e^{-\left(1/2\right)x^{2}}. \end{array}$$

For a two-dimensional case, the bivariate probability density function is calculated as a product of univariate probability functions as follows:

(4) $$\begin{array}{c} \hat{f}\left(\;x,y\;\right)=\hat{f}\left(\;x\;\right)\cdot \hat{f}\left(\;y\;\right). \end{array}$$

Here, *x* and *y* are data in each dimension, respectively.

However, each random projection produces a different mapping of the original data points which reveals only a part of the data manifold in higher-dimensional space. In case of the binary classification problem, we are interested in a mapping that separates data points belonging to two different classes best.

As a criterion for estimating the mapping, we use the Jaccard distance metric between two probability density estimates of data points representing each class. The advantage of the Jaccard distance metric as compared to other metrics of distance such as Kullback-Leibler (KL) divergence and Hellinger distance is its adaptability to multidimensional spaces where compared points show relations to different subsets. Therefore, it is well adapted to the developed model of human activity features, where according to description in the previous section we have divided them into some sets of actions. Furthermore, the computational complexity of the Hellinger distance is very high, while KL divergence might be unbounded.

The Jaccard distance, which measures dissimilarity between sample sets, is obtained by subtracting the Jaccard coefficient from 1 or, equivalently, by dividing the difference of the sizes of the union and the intersection of two sets by the size of the union:

(5) $$\begin{array}{c} d_{J}\left(\;A,B\;\right)=1-J\left(\;A,B\;\right)=\frac{\left|A\cup B\right|-\left|A\cap B\right|}{\left|A\cup B\right|}. \end{array}$$

**Figure 2:**
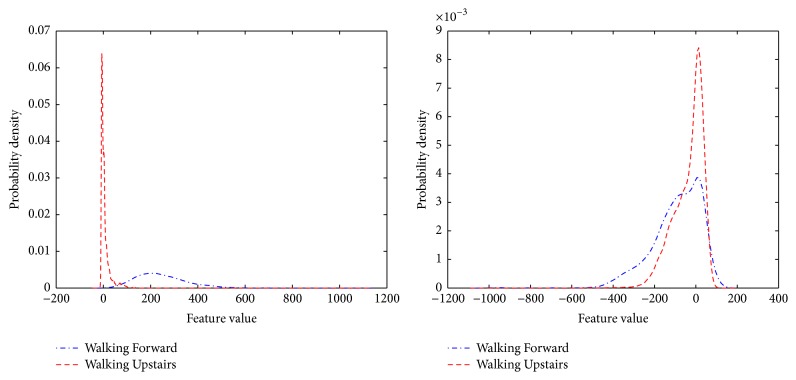
Graphical illustration of good separation versus bad separation of kernel density estimation functions (Subject 1, Trial 1, Walking Forward versus Walking Upstairs; 2nd dimension).

In the proposed model, the best random projection with the smallest overlapping area is selected (see an example in Figure 2).

**Figure 3:**
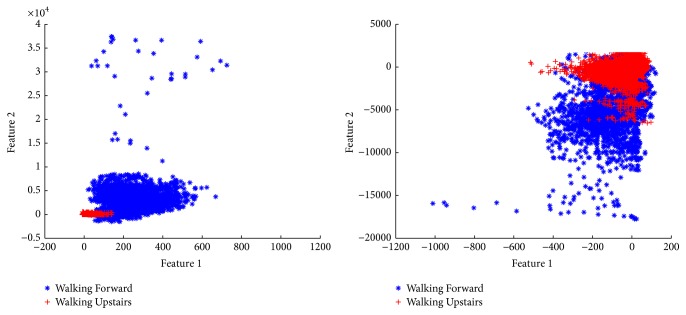
Example of classification: walking versus running (Subject 1, Trial 1) classes randomly projected in a bidimensional feature subspace.

To explore the performance and correlation among features visually, a series of scatter plots in a 2D feature space is shown in Figure 3. The horizontal and vertical axes represent two different features. The points in different colours represent different human activities.

In case of multiple classes, the method works as a one-class classifier: recognizing instances of a positive class, while all instances of other classes are recognized as outliers of the positive class.

### 3.3. Algorithm

The pseudocode of the algorithms for finding the best projection and using it for classification in low-dimensional space is presented in Pseudocodes 1 and 2, respectively.

## 4. Experiments

### 4.1. Dataset

To evaluate the performance of the proposed approach for HAR from the smartphone data, we used the part of the dataset (USC Human Activity Dataset [38]) recorded using the MotionNode device (sampling rate: 100 Hz; 3-axis accelerometer range: ±6 g; 3-axis gyroscope range: ±500 dps). The dataset consists of records recorded with 14 subjects (7 male, 7 female; age: 21–49) of 12 activities, 5 trials each. During data acquisition, MotionNode was attached on the front right hip of subjects.

The recorded low-level activities are as follows: Walking Forward (WF), Walking Left (WL), Walking Right (WR), Walking Upstairs (WU), Walking Downstairs (WD), Running Forward (RF), Jumping Up (JU), Sitting (Si), Standing (St), Sleeping (Sl), Elevator Up (EU), and Elevator Down (ED). Each record consists of the following attributes: date, subject number, age, height, weight, activity name, activity number, trial number, sensor location, orientation, and readings. Sensor readings consist of 6 readings: acceleration along *x*-, *y*-, and *z*-axes and gyroscope along *x*-, *y*-, and *z*-axes. Each trial was performed on different days at various indoor and outdoor locations.

### 4.2. Results

In Table 4, we describe the top three best features from Table 1 (see column Feature number) ranked by the Matlab* Rankfeatures* function using the* entropy* criterion.

The results of feature ranking presented in Table 5 can be summarized as follows:

(i) For Walking Forward, Walking Left, and Walking Right, the important features are moving variance of acceleration and gyroscope data, movement intensity of gyroscope data, moving variance of movement intensity of acceleration data, first eigenvalue of moving covariance between acceleration data, and polar angle of moving cumulative sum of gyroscope data.
(ii) For Walking Upstairs and Walking Downstairs, moving variance of gyroscope along *z*-axis, movement intensity of gyroscope data, and moving variance of movement intensity are the most important.
(iii) For Running Forward, moving variance of 100 samples of acceleration along *x*-axis, moving variance of 100 samples of gyroscope along *z*-axis, and moving energy of acceleration are distinguishing features.
(iv) For Jumping Up, the most important features are moving variance of acceleration, moving variance of movement intensity, and moving energy of acceleration.
(v) For Sitting, movement intensity of gyroscope data and movement intensity of difference between acceleration and gyroscope data are the most important.
(vi) For Standing, moving variance of movement intensity of acceleration data, moving variance of acceleration along *x*-axis, and first eigenvalue of moving covariance of difference between acceleration and gyroscope data are the most distinctive.
(vii) For Sleeping, the most prominent features are first eigenvalue of moving covariance between acceleration data and moving variance of movement intensity of acceleration data.
(viii) For Elevator Up and Elevator Down, the most commonly selected feature is moving variance of *z*-axis of gyroscope data. Other prominent features are first eigenvalue of moving covariance of difference between acceleration and gyroscope data and moving energy of *z*-axis of gyroscope data.

These results can be considered as consistent from what can be expected from the physical analysis of human motions in the analysed dataset.

The evaluation of HAR classification algorithms is usually made through the statistical analysis of the models using the available experimental data. The most common method is the confusion matrix which allows representing the algorithm performance by clearly identifying the types of errors (false positives and negatives) and correctly predicted samples over the test data.

The confusion matrix for within-subject activity recognition using Matlab's* Rankfeatures* is detailed in Table 5. The classification was performed using 5-fold cross-validation, using 80% of data for training and 20% of data for testing. Grand mean accuracy is 0.9552; grand mean precision is 0.9670; grand mean sensitivity is 0.9482; grand mean specificity is 0.9569; grand mean recall is 0.9482; grand mean *F*-score is 0.9482. The baseline accuracy was calculated using only the top 2 features selected by* Rankfeatures*, but without using random projections. The results show that features derived using random projections are significantly better than features derived using a common feature selection algorithm.

To take a closer look at the classification result, Table 5 shows the confusion table for classification of activities. The overall averaged recognition accuracy across all activities is 95.52%, with 11 out of 12 activities having accuracy values higher than 90%. If we examine the recognition performance for each activity individually, Running Forward, Jumping Up, and Sleeping will have very high accuracy values. For Running Forward, the accuracy of 99.0% is achieved. Interestingly, the lowest accuracy was achieved for Elevator Up activity, only 84.0%, while it was most often misclassified with Sitting and Standing. Elevator Down is misclassified with Elevator Up (only 69.7% accuracy). This result makes sense since Sitting on a chair, Standing, and Standing in a moving elevator are static activities, and we expect difficulty in differentiating different static activities. Also, there is some misclassification when deciding on a specific direction of activity; for example, Walking Left is confused with Walking Forward (77.4% accuracy) and Walking Upstairs (87.4% accuracy). Walking Upstairs is also confused with Walking Right (79.8% accuracy) and Walking Downstairs (70.8% accuracy). This is due to the similarity of any walk-related activities.

For comparison, the confusion matrix for within-subject activity recognition obtained using the proposed method with ReliefF feature selection is detailed in Table 6. The classification was performed using 5-fold cross-validation, using 80% of data for training and 20% of data for testing. Grand mean accuracy is 0.932; grand mean precision is 0.944; grand mean sensitivity is 0.939; grand mean specificity is 0.933; grand mean recall is 0.939; grand mean *F*-score is 0.922.

The baseline accuracy was calculated using only the top 2 features selected using* ReliefF*, but without using random projections. Again, the results show that features derived using random projections are significantly better than features derived using the ReliefF method only.

Surprisingly, though the classification accuracy of the specific activities differed, the mean accuracy metric results are quite similar (but still worse, if grand mean values are considered). The features identified using ReliefF feature selection were better at separating Walking Forward from Walking Left and Standing from Elevator Up activities but proved worse for separating other activities such as Sitting from Standing.

For subject identification, the data from all physical actions is used to train the classifier. Here, we consider one-versus-all subject identification problem. Therefore, the data of one subject is defined as positive class, and the data of all other subjects is defined as negative class. In this case, also 5-fold cross-validation was performed, using 80% of data for training and 20% of data for testing. The results of one-versus-all subject identification using all activities for training and testing are presented in Table 7. While the results are not very good, they still are better than random baselines: grand mean accuracy is 0.477; precision is 0.125; recall is 0.832; and *F*-score is 0.210.

If an activity of a subject has been established, separate classifiers for each activity can be used for subject identification. In this case, also 5-fold cross-validation was performed, using 80% of data for training and 20% of data for testing, and the results are presented in Table 8. The grand mean accuracy is 0.720, which is better than random baseline. However, if we consider only the top three walking-related activities (Walking Forward, Walking Left, or Walking Right), the mean accuracy is 0.944.

Finally, we can simplify the classification problem to binary classification (i.e., recognize one subject against another). This simplification can be motivated by the assumption that only a few people are living in an AAL home (far less than 14 subjects in the analysed dataset). Then, the data from a pair of subjects performing a specific activity is used for classification and training. Separate classifiers are built for each pair of subjects, the results are evaluated using 5-fold cross-validation, and the results are averaged. The results are presented in Table 9. Note that the grand mean accuracy has increased to 0.947, while, for the top three walking-related activities (Walking Forward, Walking Left, or Walking Right), the grand mean accuracy is 0.992.

## 5. Evaluation and Discussion

Random projections have been used in the HAR domain for data dimensionality reduction in activity recognition from noisy videos [69], feature compression for head pose estimation [70], and feature selection for activity motif discovery [71]. The advantages of random projections are the simplicity of their implementation and their scalability, robustness to noise, and low computational complexity: constructing the random matrix *R* and projecting the *d* × *N* data matrix into *k* dimensions are of order *O*(*dkN*).

The HAD dataset has been used in HAR research by other authors, too. Using the same HAD dataset, Zheng [66] has achieved 95.6% accuracy. He used the means and variances of magnitude and angles as the activity features and the magnitude and angles that were produced by a triaxial acceleration vector. Classifier used the Least Squares Support Vector Machine (LS-SVM) and Naïve-Bayes (NB) algorithm to distinguish different activity classes. Sivakumar [67] achieved 84.3% overall accuracy using symbolic approximation of time series of accelerometer and gyroscope signal. Vaka [68] achieved 90.7% accuracy for within-person classification and 88.6% accuracy for interperson classification using Random Forest. The features used for the recognition were time domain features: mean, standard deviation, correlation between *X* and *Y*, correlation between *Y* and  *Z*, correlation between *X* and *Z*, and root mean square of a signal. Our results (95.52% accuracy), obtained using the proposed method, are very similar to the best results of Zheng for activity recognition task.

The results obtained by different authors using the USC-HAD dataset are summarized in Table 10.

We think that it would be difficult to achieve even higher results due to some problems with the analysed dataset, which include a set of problems inherent to many Human Activity Datasets as follows:

- *Accurate Labelling of All Activities.* Existing activity recognition algorithms usually are based on supervised learning where the training data depends upon accurate labelling of all human activities. Collecting consistent and reliable data is a very difficult task since some activities may have been marked by users with wrong labels.
- *Transitionary/Overlapping Activities. *Often people do several activities at the same time. The transition states (such as walking-standing, lying-standing) can be treated as additional states, and the recognition model can be trained with respect to these states to increase the accuracy.
- *Context Problem. *It occurs when the sensors are placed at an inappropriate position relative to the activity being measured. For example, with accelerometer-based HAR, the location where the phone is carried, such as in the pocket or in the bag, impacts the classification performance.
- *Subject Sensitivity*. It measures dependency of the trained classification model upon the specifics of user.
- *Weak Link between Basic Activities and More Complex Activities. *For example, it is rather straightforward to detect whether the user is running, but inferring whether the user is running away from danger or jogging in a park is different.
- *Spurious Data.* Most published studies handle the problem of the fuzzy borders by manual data cropping.

## 6. Conclusion

Monitoring and recognizing human activities are important for assessing changes in physical and behavioural profiles of the population over time, particularly for the elderly and impaired and patients with chronic diseases. Although a wide variety of sensors are being used in various devices for activity monitoring, the positioning of the sensors, the selection of relevant features for different activity groups, and providing context to sensor measurements still pose significant research challenges.

In this paper, we have reviewed the stages needed to implement a human activity recognition method for automatic classification of human physical activity from on-body sensors. A major contribution of the paper lies in pursuing the random projections based approach for feature dimensionality reduction. The results of extensive testing performed on the USC-HAD dataset (we have achieved overall accuracy of within-person classification of 95.52% and interperson identification accuracy of 94.75%) reveal the advantages of the proposed approach. Gait-related activities (Walking Forward, Walking Left, and Walking Right) allowed the best identification of subjects opening the way for a multitude of applications in the area of gait-based identification and verification.

Future work will concern the validation of the proposed method using other datasets of human activity data as well as integration of the proposed method in the wearable sensor system we are currently developing for applications in indoor human monitoring.

## Figures and Tables

**Pseudocode 1:**
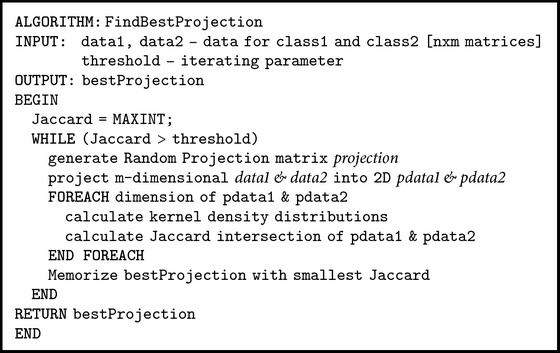
Pseudocode of FindBestProjection.

**Pseudocode 2:**
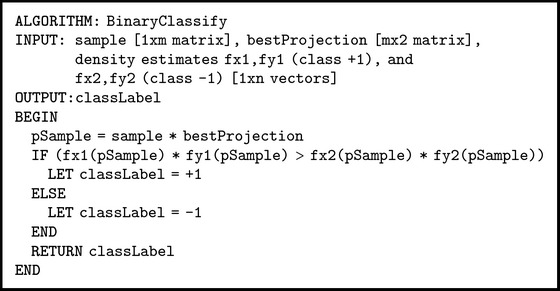
Pseudocode of binary classification.

**Table 1:** Catalogue of features.

Feature number	Description	Equation (notation)
4–6	Acceleration (*x*-, *y*-, and *z*-axes)	*a*_*x*_, *a*_*y*_, *a*_*z*_

7–9	Gyroscope (*x*-, *y*-, and *z*-axes)	*g*_*x*_, *g*_*y*_, *g*_*z*_

10–15	Moving variance of 100 samples of acceleration and gyroscope data	$var=\frac{1}{N\left(N-1\right)}\left(N\overset{N}{\underset{i=1}{\sum }}x_{i}^{2}-{\left(\overset{N}{\underset{i=1}{\sum }}x_{i}\right)}^{2}\right)$, here *x* = *a*_*x*_, *a*_*y*_, *a*_*z*_, *g*_*x*_, *g*_*y*_, *g*_*z*_

16-17	Movement intensity of acceleration and gyroscope data	${MI}_{a}=\sqrt{a_{x}^{2}+a_{y}^{2}+a_{z}^{2}}$ ${MI}_{g}=\sqrt{g_{x}^{2}+g_{y}^{2}+g_{z}^{2}}$

18	Movement intensity of difference between acceleration and gyroscope data	${MI}_{ga}=\sqrt{{\left(g_{x}-a_{x}\right)}^{2}+{\left(g_{y}-a_{y}\right)}^{2}+{\left(g_{y}-a_{y}\right)}^{2}}$

19–21	Moving variance of 100 samples of movement intensity data	$var=\frac{1}{N\left(N-1\right)}\left(N\overset{N}{\underset{i=1}{\sum }}x_{i}^{2}-{\left(\overset{N}{\underset{i=1}{\sum }}x_{i}\right)}^{2}\right)$, here *x* = MI_*a*_, MI_*g*_, MI_*ga*_

22–24	Polar coordinates of acceleration data	*φ*_*a*_ = arctan⁡(*a*_*y*_, *a*_*x*_), $r_{a}=\sqrt{a_{x}^{2}+a_{y}^{2}}$, *z*_*a*_ = *a*_*z*_

25–27	Polar coordinates of gyroscope data	*φ*_*g*_ = arctan⁡(*g*_*y*_, *g*_*x*_), $r_{g}=\sqrt{g_{x}^{2}+g_{y}^{2}}$, *z*_*g*_ = *g*_*z*_

28–30	Polar coordinates of difference between acceleration and gyroscope data	*φ*_*ag*_ = arctan⁡(*a*_*y*_ − *g*_*y*_, *a*_*x*_ − *g*_*x*_), $r_{ag}=\sqrt{{\left(a_{y}-g_{y}\right)}^{2}+{\left(a_{x}-g_{x}\right)}^{2}}$, *z*_*ag*_ = *a*_*z*_ − *g*_*z*_

31	Simple moving average of acceleration data	${SMA}_{a}=\frac{1}{N}\left(\overset{N}{\underset{i=1}{\sum }}\left|a_{x}\right|+\overset{N}{\underset{i=1}{\sum }}\left|a_{y}\right|+\overset{N}{\underset{i=1}{\sum }}\left|a_{z}\right|\right)$

32	Simple moving average of gyroscope data	${SMA}_{g}=\frac{1}{N}\left(\overset{N}{\underset{i=1}{\sum }}\left|g_{x}\right|+\overset{N}{\underset{i=1}{\sum }}\left|g_{y}\right|+\overset{N}{\underset{i=1}{\sum }}\left|g_{z}\right|\right)$

33	Simple moving average of difference between acceleration and gyroscope data	${SMA}_{ag}=\frac{1}{N}\left(\overset{N}{\underset{i=1}{\sum }}\left|a_{x}-g_{x}\right|+\overset{N}{\underset{i=1}{\sum }}\left|a_{y}-g_{y}\right|+\overset{N}{\underset{i=1}{\sum }}\left|a_{z}-g_{z}\right|\right)$

34	First eigenvalue of moving covariance between acceleration data	*E*_*a*_ = eig_1_(cov⁡(*a*_*x*_(1 : *N*), *a*_*y*_(1 : *N*), *a*_*z*_(1 : *N*)))

35	First eigenvalue of moving covariance between gyroscope data	*E*_*g*_ = eig_1_(cov⁡(*g*_*x*_(1 : *N*), *g*_*y*_(1 : *N*), *g*_*z*_(1 : *N*)))

36	First eigenvalue of moving covariance of difference between acceleration and gyroscope data	*E*_*ag*_ = eig_1_(cov(*a*_*x*_ − *g*_*x*_, *a*_*y*_ − *g*_*y*_, *a*_*z*_ − *g*_*z*_))

37–42	Moving energy of acceleration and gyroscope data	$ME=\frac{1}{N}\overset{N}{\underset{i=1}{\sum }}x_{i}^{2}$, here *x* = *a*_*x*_, *a*_*y*_, *a*_*z*_, *g*_*x*_, *g*_*y*_, *g*_*z*_

43–48	Difference between moving maximum and moving minimum of acceleration and gyroscope data	$MinMax=\underset{1\leq i\leq N}{max}\left(x_{i}\right)-\underset{1\leq i\leq N}{min}\left(x_{i}\right)$, here *x* = *a*_*x*_, *a*_*y*_, *a*_*z*_, *g*_*x*_, *g*_*y*_, *g*_*z*_

49	Moving correlation between *x*- and *y*-axis of acceleration data	MC_*a*_^*xy*^ = corr(*a*_*x*_, *a*_*y*_)

50	Moving correlation between *x*-axis and *z*-axis of acceleration data	MC_*a*_^*xz*^ = corr(*a*_*x*_, *a*_*z*_)

51	Moving correlation between *y*-axis and *z*-axis of acceleration data	MC_*a*_^*yz*^ = corr(*a*_*y*_, *a*_*z*_)

52	Moving correlation between *x*-axis and *y*-axis of gyroscope data	MC_*g*_^*xy*^ = corr(*g*_*x*_, *g*_*y*_)

53	Moving correlation between *x*-axis and *z*-axis of gyroscope data	MC_*g*_^*xz*^ = corr(*g*_*x*_, *g*_*z*_)

54	Moving correlation between *y*-axis and *z*-axis of gyroscope data	MC_*g*_^*yz*^ = corr(*g*_*y*_, *g*_*z*_)

55–57	Projection of gyroscope data onto acceleration data	$P=a-\frac{\left(g^{T}ag\right)}{{\left|g\right|}^{2}}$

58	Moving mean of orientation vector of acceleration data	$MMA=\frac{1}{N}\overset{N}{\underset{i=1}{\sum }}\varphi_{i}$, here $\varphi =\frac{\arccos\left(a_{x}\cdot a_{y}\right)}{\left|a_{x}\right|\cdot \left|a_{y}\right|}$

59	Moving variance of orientation vector of acceleration data	$MVA=\frac{1}{N\left(N-1\right)}\left({\left(\overset{N}{\underset{i=1}{\sum }}\varphi_{i}\right)}^{2}-\overset{N}{\underset{i=1}{\sum }}\varphi_{i}^{2}\right)$, $\varphi =\frac{\arccos\left(a_{x}\cdot a_{y}\right)}{\left|a_{x}\right|\cdot \left|a_{y}\right|}$

60	Moving energy of orientation vector of acceleration data	$MEA=\frac{1}{N}\overset{N}{\underset{i=1}{\sum }}\varphi_{i}^{2}$, here $\varphi =\frac{\arccos\left(a_{x}\cdot a_{y}\right)}{\left|a_{x}\right|\cdot \left|a_{y}\right|}$

61–63	Moving energy of difference between acceleration and gyroscope data	${ME}_{ag}=\frac{1}{N}\overset{N}{\underset{i=1}{\sum }}{\left(x_{i}-y_{i}\right)}^{2}$, here *x* = *a*_*x*_, *a*_*y*_, *a*_*z*_, *y* = *g*_*x*_, *g*_*y*_, *g*_*z*_

64	Moving energy of difference between *x*-axis and *y*-axis of acceleration data	${ME}_{xy}=\frac{1}{N}\overset{N}{\underset{i=1}{\sum }}{\left(a_{x,i}-a_{y,i}\right)}^{2}$

65	Moving energy of difference between *x*-axis and *z*-axis of acceleration data	${ME}_{xz}=\frac{1}{N}\overset{N}{\underset{i=1}{\sum }}{\left(a_{x,i}-a_{z,i}\right)}^{2}$

66	Moving energy of difference between *y*-axis and *z*-axis of acceleration data	${ME}_{yz}=\frac{1}{N}\overset{N}{\underset{i=1}{\sum }}{\left(a_{y,i}-a_{z,i}\right)}^{2}$

67	Moving mean of orientation vector of difference between acceleration and gyroscope data	$MMA=\frac{1}{N}\overset{N}{\underset{i=1}{\sum }}\varphi_{i}$, here $\varphi =\frac{\arccos\left(\left(a_{x}-g_{x}\right)\left(a_{y}-g_{y}\right)\right)}{\left|a_{x}-g_{x}\right|\cdot \left|a_{y}-g_{y}\right|}$

68	Moving variance of orientation vector of difference between acceleration and gyroscope data	$MVA=\frac{1}{N\left(N-1\right)}\left({\left(\overset{N}{\underset{i=1}{\sum }}\varphi_{i}\right)}^{2}-\overset{N}{\underset{i=1}{\sum }}\varphi_{i}^{2}\right)$, $\varphi =\frac{\arccos\left(\left(a_{x}-g_{x}\right)\left(a_{y}-g_{y}\right)\right)}{\left|a_{x}-g_{x}\right|\cdot \left|a_{y}-g_{y}\right|}$

69	Moving energy of orientation vector of difference between acceleration and gyroscope data	$MEA=\frac{1}{N}\overset{N}{\underset{i=1}{\sum }}\varphi_{i}^{2}$, here $\varphi =\frac{\arccos\left(\left(a_{x}-g_{x}\right)\left(a_{y}-g_{y}\right)\right)}{\left|a_{x}-g_{x}\right|\cdot \left|a_{y}-g_{y}\right|}$

70	Moving mean of orientation vector of gravity data	${MMA}_{g}=\frac{1}{N}\overset{N}{\underset{i=1}{\sum }}\varphi_{i}$, here $\varphi =\frac{\arccos\left(g_{x}\cdot g_{y}\right)}{\left|g_{x}\right|\cdot \left|g_{y}\right|}$

71	Moving variance of orientation vector of gravity data	${MVA}_{g}=\frac{1}{N\left(N-1\right)}\left({\left(\overset{N}{\underset{i=1}{\sum }}\varphi_{i}\right)}^{2}-\overset{N}{\underset{i=1}{\sum }}\varphi_{i}^{2}\right)$, $\varphi =\frac{\arccos\left(g_{x}\cdot g_{y}\right)}{\left|g_{x}\right|\cdot \left|g_{y}\right|}$

72	Moving energy of orientation vector of gravity data	${MEA}_{g}=\frac{1}{N}\overset{N}{\underset{i=1}{\sum }}\varphi_{i}^{2}$, here $\varphi =\frac{\arccos\left(g_{x}\cdot g_{y}\right)}{\left|g_{x}\right|\cdot \left|g_{y}\right|}$

73	Moving mean of orientation vector of difference between acceleration and gravity data	${MMA}_{ag}=\frac{1}{N}\overset{N}{\underset{i=1}{\sum }}\varphi_{i}$, $\varphi =\frac{\arccos\left(a_{x}\cdot a_{y}\right)}{\left|a_{x}\right|\cdot \left|a_{y}\right|}-\frac{\arccos\left(g_{x}\cdot g_{y}\right)}{\left|g_{x}\right|\cdot \left|g_{y}\right|}$

74	Moving variance of orientation vector of difference between acceleration and gravity data	${MVA}_{ag}=\frac{1}{N\left(N-1\right)}\left({\left(\overset{N}{\underset{i=1}{\sum }}\varphi_{i}\right)}^{2}-\overset{N}{\underset{i=1}{\sum }}\varphi_{i}^{2}\right)$, $\varphi =\frac{\arccos\left(a_{x}\cdot a_{y}\right)}{\left|a_{x}\right|\cdot \left|a_{y}\right|}-\frac{\arccos\left(g_{x}\cdot g_{y}\right)}{\left|g_{x}\right|\cdot \left|g_{y}\right|}$

75	Moving energy of orientation vector of difference between acceleration and gravity data	${MEA}_{ag}=\frac{1}{N}\overset{N}{\underset{i=1}{\sum }}\varphi_{i}^{2}$, $\varphi =\frac{\arccos\left(a_{x}\cdot a_{y}\right)}{\left|a_{x}\right|\cdot \left|a_{y}\right|}-\frac{arccos\left(g_{x}\cdot g_{y}\right)}{\left|g_{x}\right|\cdot \left|g_{y}\right|}$

76–81	Moving cumulative sum of acceleration and gyroscope data	$MCS=\overset{N}{\underset{j=1}{\sum }}\overset{j}{\underset{i=1}{\sum }}x_{i}$, here *x* = *a*_*x*_, *a*_*y*_, *a*_*z*_, *g*_*x*_, *g*_*y*_, *g*_*z*_

82	Simple moving average of cumulative sums of acceleration data	${SMAMCS}_{a}=\frac{1}{N}\overset{N}{\underset{i=1}{\sum }}{MCS}_{a,i}$

83	Simple moving average of cumulative sums of gyroscope data	${SMAMCS}_{g}=\frac{1}{N}\overset{N}{\underset{i=1}{\sum }}{MCS}_{g,i}$

84	Simple moving average of cumulative sums of difference between accelerometer and gyroscope data	${SMAMCS}_{ag}=\frac{1}{N}\overset{N}{\underset{i=1}{\sum }}\left({MCS}_{a,i}-{MCS}_{g,i}\right)$

85–90	Moving 2nd-order cumulative sum of acceleration and gyroscope data	${MCS}^{\prime }=\overset{N}{\underset{j=1}{\sum }}\overset{j}{\underset{i=1}{\sum }}{MCS}_{i}$

91–93	Moving 2nd-order cumulative sum of differences between cumulative sums of acceleration and gyroscope data	${{MCS}^{\prime }}_{ag}=\overset{N}{\underset{j=1}{\sum }}\overset{j}{\underset{i=1}{\sum }}\left({MCS}_{i}^{a}-MCS_{i}^{g}\right)$

94–96	Polar coordinates of moving cumulative sum of acceleration data	*φ*′_*a*_ = arctan⁡(MCS_*y*_^*a*^, MCS_*x*_^*a*^), ${r^{\prime }}_{a}=\sqrt{{\left({MCS}_{x}^{a}\right)}^{2}+{\left({MCS}_{y}^{a}\right)}^{2}}$, *z*′_*a*_ = MCS_*z*_^*a*^

97–99	Polar coordinates of moving cumulative sum of gyroscope data	*φ*′_*g*_ = arctan⁡(MCS_*y*_^*g*^, MCS_*x*_^*g*^), ${r^{\prime }}_{g}=\sqrt{{\left({MCS}_{x}^{g}\right)}^{2}+{\left({MCS}_{y}^{g}\right)}^{2}}$, *z*′_*g*_ = MCS_*z*_^*g*^

100–102	Polar coordinates of moving cumulative sum of differences between acceleration and gyroscope data	*φ*′_*ag*_ = arctan⁡(MCS_*y*_^*a*^ − MCS_*y*_^*g*^, MCS_*x*_^*a*^ − MCS_*x*_^*g*^), ${r^{\prime }}_{ag}=\sqrt{{\left({MCS}_{x}^{a}-{MCS}_{x}^{g}\right)}^{2}+{\left({MCS}_{y}^{a}-{MCS}_{y}^{g}\right)}^{2}}$, *z*′_*ag*_ = MCS_*z*_^*a*^ − MCS_*z*_^*g*^

**Table 2:** Summary of feature selection/dimensionality reduction methods in HAR.

Method	Advantages	Disadvantages	Complexity
PCA	High dimensionality reduction; reduction of noise; lack of redundancy of data due to orthogonality of components	The covariance matrix is difficult to be evaluated accurately; even the simplest invariance could not be captured by the PCA unless the training data explicitly provides for it	*O*(*p* ^2^ *n* + *p* ^3^), where *n* are data points, each represented with *p* features

*ReliefF*	Low computational complexity	Unstable due to random selection of instances	*O*(*p* · *n* · log⁡*n*)

Rankfeatures	Features highly correlated with already selected features are less likely to be included	It assumes that data classes are normally distributed	It depends upon class separability criterion

CFS	It evaluates a subset of features rather than individual features	It fails to select locally predictive features when they are overshadowed by strong, globally predictive features	$O\left(n\left(\frac{p^{2}-p}{2}\right)\right)$

**Table 3:** Summary of related works in the HAR domain.

Author	Activities	Sensor data	Features	Feature selection	Classification method	Accuracy
Atallah et al. [39]	Lying down, preparing food, eating and drinking, socialising, reading, dressing, walking, treadmill walking, vacuuming, wiping tables, running, treadmill, running, cycling, sitting down/getting up, and lying down/getting up	Acceleration sensors	Averaged entropy over 3 axes, main FFT frequency (averaged) over 3 axes, energy of the 0.2 Hz window centred around main frequency over total FFT energy (3-axis average), and averaged mean of cross covariance between every 2 axes	*ReliefF*, Simba, and MRMR	kNN, Bayesian classifier	90%

Bayat et al. [40]	Running, slow walk, fast walk, aerobic dancing, stairs up, and stairs down	Triaxial accelerometer	Mean along *z*-axis, MinMax, STD, and RMS for Am, APF along *x*-axis, *y*-axis, and *z*-axis, VarAPF, STD along *x*-axis, *y*-axis, and *z*-axis, RMS along *x*-axis, *y*-axis, and *z*-axis, correlation between *z*-axis and *y*-axis, and MinMax along *x*-axis, *y*-axis, and *z*-axis	Feature clustering	Multilayer perceptron, SVM, Random Forest, and Logit Boost	81%–91%

Berchtold et al. [41]	Standing, sitting, lying, walking, climbing stairs, cycling, and being stationary	Accelerometer	Variance, mean	None	Fuzzy inference	97.3%

Capela et al. [17]	Sitting, standing, and lying; ramp up and ramp down; stairs up and stairs down; transition between activities	Linear acceleration, gravity, and velocity sensors	Range, mean, standard deviation, kurtosis, moving average, covariance matrix, skewness, zero cross rate, and mean cross rate	None	Naïve-Bayes, Support Vector Machine, and j48 decision tree	97%

Gupta and Dallas [30]	Jumping, running, walking, sitting, sitting-to-standing, and standing-to-kneeling	Triaxial accelerometer	Energy, entropy, mean, variance, mean trend, windowed mean difference, variance trend, windowed variance difference, detrended fluctuation analysis coefficients, *X*-*Z*-energy, and max. difference acceleration	*ReliefF*, SFFS	kNN, Naive Bayes	98%

Henpraserttae et al. [42]	Sitting, lying, standing, and walking	Accelerometer	Mean and standard deviation	None	Rules and threshold based classification	90%

Hoque and Stankovic [43]	Leaving house, using toilet, taking shower, sleeping, preparing breakfast, preparing dinner, getting snack, getting drink, using washing machine, and using dishwasher	Location sensors (open/closed)	Magnitude	None	Custom clustering method	64.5%–89.9%

Iso and Yamazaki [44]	Walking, running, stairs up/down, and fast walking	Accelerometer	Wavelet components, periodograms, and information entropy	None	Bayesian probabilities	80%

Kose et al. [45]	Walking, running, biking, sitting, and standing	Accelerometer	Min., max., average, variance, FFT coefficients, and autocorrelation	None	Clustered kNN	95.2%–97.5%

Kwapisz et al. [46]	Walking, jogging, stairs up/down, sitting, and standing	Accelerometer	Mean, std. dev., average absolute difference, average resultant acceleration, time between peaks, and binned distribution	None	Decision tree, logistic regression, and MNN	91.7%

Lane et al. [47]	Driving, being stationary, running, and walking	GPS, accelerometer, and microphone	Mean, variance	None	Naïve-Bayes	85–98%

Lee and Cho [48]	Standing, walking, running, stairs up/down, shopping, and taking bus	Accelerometer	*x*-, *y*-, and *z*-axes acceleration values	None	Hierarchical HMM	70%–90%

Mannini and Sabatini [49]	Walking, walking carrying items, sitting & relaxing, working on computer, standing still, eating or drinking, watching TV, reading, running, bicycling, stretching, strength training, scrubbing, vacuuming, folding laundry, lying down and relaxing, brushing teeth, climbing stairs, riding elevator, and riding escalator	Acceleration sensors	DC component, energy, frequency-domain entropy, and correlation coefficients	SFFS (Pudil algorithm)	Continuous emissions, Hidden Markov Model	99.1%

Mathie et al. [37]	Various human movements, including resting, walking, and falling	Triaxial acceleration sensor	Integrated area under curve	None	Binary decision tree	97.7% (sensitivity) 98.7% (specificity)

Maurer et al. [50]	Walking, standing, sitting, running, and ascending and descending the stairs	Multiple sensors	Mean, root mean square, standard deviation, variance, mean absolute deviation, cumulative histogram, *n*th percentiles, interquartile range, zero crossing rate, mean crossing rate, and sq. length of *X*, *Y*	Correlation-based Feature Selection (CFS)	Decision trees (C4.5 algorithm), *k*-Nearest Neighbor, Naïve-Bayes, and Bayes Net	80%–92%

Miluzzo et al. [51]	Sitting, standing, walking, and running	Accelerometer, GPS, and audio	DFT, FFT features, mean, std. dev. and number of peaks per unit, and time deviation of DFT power	None	Decision tree	79%

Pärkkä et al. [52]	Lying down, rowing, ex-biking, sitting/standing, running, and Nordic walking	GPS, audio, altitude, EKG, accelerometer, compass, humidity, light, temperature, heart rate, pulse, respiratory effort, and skin resistance	Peak frequency of up-down chest acceleration, median of up-down chest acceleration, peak power of up-down chest acceleration, variance of back-forth chest acceleration, sum of variances of 3D wrist acceleration, and power ratio of frequency bands 1–1.5 Hz and 0.2–5 Hz measured from chest magnetometer	Heuristic	Decision tree	86%

Saponas et al. [53]	Walking, jogging	Accelerometer	124 features: Nike + iPod Packet Payload, magnitude (mean, std. dev., min., max., and min. minus max.), frequency (energy in each of the first 10 frequency components of DFT, energy in each band of 10 frequency components, largest frequency component, and index of the largest frequency component)	None	Naïve-Bayesian Network	97.4% (within-person), 99.48% (cross-person)

Siirtola and Röning [54]	Walking, running, cycling, driving, sitting, and standing	Accelerometer	Magnitude, std., mean, min., max., percentiles (10, 25, 50, 75, and 90), and sum and square sum of observations above/below percentile (5, 10, 25, 75, 90, and 95) of magnitude acceleration and square sum of *x* & *z*	None	Decision tree + kNN/QDA	95%

Sohn et al. [55]	Walking, driving, and dwelling	GPS	Spearman rank correlation, variance, and mean Euclidean distance over a window of measurements	None	Logistic regression	85%

Yang [56]	Sitting, Standing, walking, running, driving, and bicycling	Accelerometer	Mean, std., zero crossing rate, 75th percentile, interquartile, spectrum centroid, entropy, and cross-correlation	None	Decision tree, Naïve-Bayes, kNN, and SVM	90%

Zhu and Sheng [57]	Sitting, standing, lying, walking, sitting-to-standing, standing-to-sitting, lying-to-sitting, and sitting-to-lying	3D acceleration	Mean, variance	None	Neural network ensemble	67%–98%

**Table 4:** Top features for binary classification of human activities.

Activity	WL	WR	WU	WD	RF	JU	Si	St	Sl	EU	ED
WF	97, 88, 91	85, 100, 88	15, 63, 42	14, 39, 34	10, 15, 60	10, 19, 37	17, 18, 30	19, 36, 35	34, 19, 36	15, 36, 35	15, 42, 63
WL		97, 91, 88	97, 63, 42	87, 97, 86	10, 37, 19	10, 37, 19	17, 18, 30	19, 36, 34	34, 19, 36	15, 36, 35	15, 42, 63
WR			63, 42, 15	34, 87, 39	10, 59, 37	10, 37, 19	17, 18, 30	19, 34, 14	34, 19, 85	15, 36, 35	15, 42, 63
WU				87, 39, 78	63, 42, 15	10, 19, 63	17, 18, 26	19, 14, 62	34, 19, 35	15, 36, 35	15, 42, 63
WD					65, 38, 34	10, 19, 60	17, 18, 7	19, 10, 20	19, 34, 35	15, 36, 35	15, 42, 63
RF						35, 36, 62	38, 36, 17	19, 10, 15	34, 19, 35	15, 36, 35	15, 42, 63
JU							4, 22, 16	19, 10, 15	19, 34, 36	15, 42, 63	15, 42, 63
Si								22, 5, 38	22, 4, 85	15, 42, 63	59, 60, 15
St									76, 85, 39	10, 15, 36	59, 11, 60
Sl										15, 22, 42	59, 60, 94
EU											59, 60, 10

WF: Walking Forward; WL: Walking Left; WR: Walking Right; WU: Walking Upstairs; WD: Walking Downstairs; RF: Running Forward; JU: Jumping Up; Si: Sitting; St: Standing; Sl: Sleeping; EU: Elevator Up; ED: Elevator Down.

**Table 5:** The confusion matrix of within-subject activity classification using *Rankfeatures*.

Activity	WF	WL	WR	WU	WD	RF	JU	Si	St	Sl	EU	ED
WF	1	0.774	0.980	0.874	0.985	0.996	0.980	0.997	0.999	1	0.999	1
WL	0.774	1	0.989	0.968	0.958	0.998	0.996	0.951	0.999	1	0.999	1
WR	0.980	0.989	1	0.798	0.998	0.997	0.988	0.988	0.971	1	0.981	1
WU	0.874	0.968	0.798	1	0.708	0.985	0.979	0.962	0.998	1	0.971	1
WD	0.985	0.958	0.998	0.708	1	0.992	0.878	0.967	0.850	1	0.986	1
RF	0.996	0.998	0.997	0.985	0.992	1	0.978	0.991	0.996	0.957	0.994	1
JU	0.980	0.996	0.988	0.979	0.878	0.978	1	0.973	1	0.929	1	1
Si	0.997	0.951	0.988	0.962	0.967	0.991	0.973	1	0.987	1	0.126	0.992
St	0.999	0.999	0.971	0.999	0.850	0.995	1	0.987	1	1	0.326	0.887
Sl	1	1	1	1	1	0.957	0.930	1	1	1	1	0.992
EU	0.999	0.999	0.981	0.971	0.986	0.994	1	0.126	0.326	1	1	0.697
ED	1	1	1	1	1	1	1	0.992	0.887	0.992	0.697	1
Mean	**0.965**	**0.969**	**0.974**	**0.937**	**0.944**	**0.990**	**0.975**	**0.911**	**0.918**	**0.989**	**0.840**	**0.964**
Baseline	0.650	0.616	0.616	0.712	0.713	0.621	0.641	0.627	0.642	0.651	0.640	0.628

WF: Walking Forward; WL: Walking Left; WR: Walking Right; WU: Walking Upstairs; WD: Walking Downstairs; RF: Running Forward; JU: Jumping Up; Si: Sitting; St: Standing; Sl: Sleeping; EU: Elevator Up; ED: Elevator Down.

**Table 6:** The confusion matrix of within-subject activity classification using *ReliefF*.

Activity	WF	WL	WR	WU	WD	RF	JU	Si	St	Sl	EU	ED
WF	1.000	0.998	0.892	0.931	0.993	1.000	0.999	1.000	1.000	1.000	1.000	1.000
WL	0.998	1.000	0.853	0.989	0.997	1.000	1.000	0.999	1.000	0.896	0.971	1.000
WR	0.892	0.853	1.000	0.789	0.964	1.000	1.000	0.999	1.000	0.853	1.000	1.000
WU	0.931	0.989	0.789	1.000	0.702	1.000	1.000	0.996	0.956	0.992	0.999	1.000
WD	0.993	0.997	0.964	0.702	1.000	0.965	0.998	0.655	0.997	0.982	1.000	0.975
RF	1.000	1.000	1.000	1.000	0.965	1.000	0.688	0.999	1.000	1.000	1.000	1.000
JU	0.999	1.000	1.000	1.000	0.998	0.688	1.000	1.000	1.000	0.993	1.000	1.000
Si	1.000	0.999	0.999	0.996	0.655	0.999	1.000	1.000	0.491	0.967	0.328	0.313
St	1.000	1.000	1.000	0.956	0.997	1.000	1.000	0.491	1.000	0.766	0.528	0.901
Sl	1.000	0.896	0.853	0.992	0.982	1.000	0.993	0.967	0.766	1.000	1.000	1.000
EU	1.000	0.971	1.000	0.999	1.000	1.000	1.000	0.328	0.528	1.000	1.000	0.765
ED	1.000	1.000	1.000	1.000	0.975	1.000	1.000	0.313	0.901	1.000	0.765	1.000
Mean	**0.984**	**0.975**	**0.946**	**0.946**	**0.936**	**0.971**	**0.973**	**0.812**	**0.887**	**0.954**	**0.883**	**0.913**
Baseline	0.621	0.637	0.600	0.695	0.703	0.644	0.618	0.628	0.640	0.644	0.635	0.642

WF: Walking Forward; WL: Walking Left; WR: Walking Right; WU: Walking Upstairs; WD: Walking Downstairs; RF: Running Forward; JU: Jumping Up; Si: Sitting; St: Standing; Sl: Sleeping; EU: Elevator Up; ED: Elevator Down.

**Table 7:** Results of one-versus-all subject identification (all activities).

Subjects	Accuracy	Precision	Recall	*F*-score
S1	0.657	0.142	0.744	0.239
S2	0.273	0.075	0.917	0.139
S3	0.549	0.078	0.716	0.140
S4	0.496	0.073	0.697	0.132
S5	0.323	0.068	0.931	0.127
S6	0.863	0.220	0.637	0.328
S7	0.265	0.055	0.920	0.103
S8	0.107	0.071	0.985	0.132
S9	0.683	0.229	0.967	0.370
S10	0.156	0.091	0.943	0.166
S11	0.755	0.263	0.905	0.407
S12	0.689	0.167	0.533	0.254
S13	0.373	0.115	0.881	0.203
S14	0.493	0.112	0.866	0.198
Mean	**0.477**	**0.123**	**0.832**	**0.210**
Random baseline	0.071	0.071	0.929	0.133

**Table 8:** Results of one-versus-all subject identification for specific activities.

Activity	Accuracy	*F*
Walking Forward	0.947	0.727
Walking Left	0.955	0.769
Walking Right	0.931	0.722
Walking Upstairs	0.857	0.551
Walking Downstairs	0.833	0.497
Running Forward	0.832	0.496
Jumping Up	0.814	0.453
Sitting	0.506	0.391
Standing	0.722	0.432
Sleeping	0.589	0.292
Elevator Up	0.337	0.235
Elevator Down	0.318	0.232
Mean	**0.720**	**0.483**
Random baseline	0.071	0.133

**Table 9:** Accuracy of binary subject identification using separate activities.

Activity	Accuracy	*F*
Walking Forward	0.992	0.987
Walking Left	0.989	0.987
Walking Right	0.993	0.993
Walking Upstairs	0.977	0.970
Walking Downstairs	0.974	0.971
Running Forward	0.980	0.974
Jumping Up	0.983	0.980
Sitting	0.883	0.859
Standing	0.940	0.932
Sleeping	0.956	0.953
Elevator Up	0.856	0.847
Elevator Down	0.846	0.822
Mean	**0.947**	**0.939**
Random baseline	0.5	0.5

**Table 10:** Summary of HAR results using USC-HAD dataset.

Reference	Features	Classification method	Accuracy
Zheng [66]	Means and variances of magnitude and angles of acceleration along *x*-, *y*- & *z*-axes	Least Squares Support Vector Machine (LS-SVM), Naïve-Bayes (NB)	95.6%
Sivakumar [67]	Accelerometer and gyroscope data	Symbolic approximation	84.3%
Vaka [68]	Mean, std. dev., correlation between *X* & *Y*, *Y* & *Z*, and *X* & *Z*, and RMS	Random Forest	90.7%
This paper	99 times, frequency and physical features	Heuristic (random projections + PDFs + Jaccard distance)	95.52%
